# Types of naming errors in chronic post-stroke aphasia are dissociated by dual stream axonal loss

**DOI:** 10.1038/s41598-018-32457-4

**Published:** 2018-09-25

**Authors:** Emilie T. McKinnon, Julius Fridriksson, Alexandra Basilakos, Gregory Hickok, Argye E. Hillis, M. Vittoria Spampinato, Ezequiel Gleichgerrcht, Chris Rorden, Jens H. Jensen, Joseph A. Helpern, Leonardo Bonilha

**Affiliations:** 1 Department of Neurology, Medical University of South Carolina, 96 Jonathan Lucas St, Charleston, SC 29425 USA; 2 Center for Biomedical Imaging, Medical University of South Carolina, 96 Jonathan Lucas St, Charleston, SC 29425 USA; 3 Department of Neuroscience, Medical University of South Carolina, 173 Ashley Avenue, Charleston, SC 29425 USA; 4 Department of Communication Sciences and Disorders, University of South Carolina, 921 Assembly Street, Columbia, SC 29208 USA; 5 Department of Cognitive Sciences, Center for Language Science and Center for Cognitive Neuroscience, University of California, 2201 Social & Behavioral Sciences Gateway Building, Irvine, CA 92697 USA; 6 Department of Neurology, Johns Hopkins University, 725 N Wolfe St, Baltimore, MD 21205 USA; 7 Department of Radiology and Radiological Science, Medical University of South Carolina, 96 Jonathan Lucas St, Charleston, SC 29425 USA; 8 Department of Psychology, University of South Carolina, 1512 Pendelton Street, Columbia, SC 29208 USA

## Abstract

The types of errors during speech production can vary across individuals with chronic post-stroke aphasia, possibly due to the location and extent of brain damage. In this study, we evaluated the relationship between semantic vs. phonemic errors during confrontational naming, and their relationship with the degree of damage to ventral and dorsal white matter pathways extending beyond the necrotic stroke lesion. Based on the dual stream model of language processing, we tested the hypothesis that semantic errors would be associated with ventral stream damage, whereas phonemic errors would be associated with dorsal stream damage, but not vice-versa. Multi-shell diffusion MRI was used to obtain kurtosis-based white matter tractography from 32 chronic stroke survivors. Using diffusion microstructural tissue modeling, we estimated axonal loss along the length of the inferior and superior longitudinal fasciculi (ILF and SLF), representing the main pathways in the ventral and dorsal streams, respectively. The frequency of semantic paraphasias was strongly associated with ILF axonal loss, whereas phonemic paraphasias were strongly associated with SLF axonal loss, but not vice versa. This dissociation between semantic and phonological processing is in agreement with the dual stream model of language processing and corroborates the concept that, during speech production, knowledge association (semantics) depends on the integrity of ventral, whereas form encoding (phonological encoding) is more localized to dorsal pathways. These findings also demonstrate the importance of the residual integrity of specific white matter pathways beyond regional gray matter damage for speech production.

## Introduction

Many stroke survivors experience language impairments (aphasia) beyond six months after a dominant hemisphere stroke^1^. One of the most common and debilitating impairments in individuals with chronic aphasia is the inability to accurately produce language, commonly represented by difficulties in naming objects or actions (anomia)^2–4^. Naming is often assessed in the clinical setting through confrontational naming tests, during which the individual with anomia is either unable to produce the correct name for a target item, or generates words that are related in sound or meaning with the target (paraphasias). Paraphasias are erroneous attempts that relate to the target, but are inaccurate regarding the chosen speech units (phonemic paraphasias), or are real words that relate in meaning to the intended word (semantic paraphasias). For example, when shown a picture of a “pencil”, a phonemic paraphasia could be an utterance such as “wencil”, whereas a semantic paraphasia could be “pen”.

Paraphasias offer a critical window into the mechanisms of speech production because they represent discrete deficits regarding (1) the spoken sound structure (phonemic) vs. (2) speech related knowledge association (semantic). They provide the opportunity to determine if these processes dissociate into different anatomic-functional pathways. Notably, they permit the assessment of a recent neurolinguistic theory, the dual stream model of speech processing, which suggests that distinct anatomical streams map phonological and lexical-semantic content retrieval during speech processing^5^. The model suggests the existence of two streams: a ventral, or “what” stream, which maps between lexical and semantic representations of knowledge associations, and a dorsal, or “how” stream, that maps between auditory and articulatory-motor representations for phonological production. In the context of a naming task, both networks are assumed to participate at integrated different levels of the naming process, lexical-semantic (ventral networks) and phonological encoding (dorsal networks). Paraphasias have been explored in the context of the dual stream model using computer simulations of speech data^6^, lesion symptom mapping^7–13^, and direct electrical cortical and subcortical stimulation in the intra-operative setting^14^. However, the relationship between residual white matter network integrity, subcortical networks (specifically in relationship to core tracts in the ventral and dorsal streams), and speech production errors is not yet fully defined.

Diffusion MRI (dMRI) is ideally suited to study post-stroke residual white matter integrity non-invasively and to test the hypothesis that semantic and phonological processing dissociates between the ventral and dorsal white matter pathways. Specifically, dMRI permits the estimation of the remaining anatomical connections between brain regions through tractography, as well as an assessment of the microstructural integrity of these connections. The quantification of pathway-specific white matter integrity in stroke survivors with chronic aphasia allows for the evaluation of the relationship between regional white matter integrity and specific types of language deficits. This process is analogous to classical neuropsychological approaches which relate brain lesions to behavioral deficits, but it can leverage white matter tractography to identify residual pathways associated with language processing. Moreover, since dMRI also provides measures sensitive to axonal integrity, it can improve the sensitivity in determining which pathways are associated with phonological vs. semantic deficits and resolve the underlying neurobiological mechanisms of regional brain damage that contribute to speech production errors. In contrast with lesion-symptom mapping, which can provide information on damage to white matter regions, without directly testing the integrity of specific pair-wise connections, dMRI can provide information on the integrity and microstructural properties of residual regional connections.

The axonal water fraction (AWF), i.e., the ratio of intra-axonal water to total MRI-visible water, reflects axonal density by assessing the portion of tissue water that resides inside axons. Multi-shell dMRI permits an estimation of AWF through the calculation of the diffusional kurtosis by fitting a second-order Taylor expansion to the decay of the logarithm of the dMRI signal as a function of diffusion weighting strength following the theory for diffusional kurtosis imaging (DKI)^15,16^. Fractional anisotropy (FA) is a dMRI measure more commonly used to investigate white matter microstructure^17^. However, FA is a generic indicator of diffusion anisotropy^18^. AWF can indicate brain damage through axonal loss^19^ and provides information complementary to FA that allows for a more comprehensive quantification of brain tissue properties. More microstructure specific diffusion metrics (e.g. AWF) should be used in conjunction with more traditional metrics (e.g. FA, MD) to enhance our understanding of the mechanisms underlying FA and MD changes, and ultimately brain pathology.

In this study, we examined whether the degree of ventral vs. dorsal stream damage dissociates the proportion of phonemic and semantic paraphasias in a group of individuals with chronic aphasia. We employed multi-shell dMRI, DKI post-processing and the quantification of AWF and FA along stream-specific fiber pathways obtained from each individual with aphasia. Tract-specific measures were assessed in relation with paraphasias using multivariate statistical analyses. We hypothesized that in chronic stroke axonal loss, reflected by a decreased AWF in the ventral but not dorsal stream, would directly associate with semantic but not phonological paraphasias and vice versa.

## Materials and Methods

### Subjects

We studied 32 participants with chronic post-stroke aphasia (age: 57 ± 11 y, time post-stroke: 35 ± 30 mos, gender: 8 women). All subjects were recruited through local advertisement at the Medical University of South Carolina. They were right-handed native English speakers with a history of a single previous ischemic stroke in the left hemisphere at least 6 months before enrollment. Demographical and behavioral information is presented in Supplementary Table S1. Participants with a history of developmental language disorders, other neurological or psychiatric problems, brain surgery or with seizures during the previous 12 months were excluded. This study was approved by the Institutional Review Boards at the Medical University of South Carolina and the University of South Carolina. Written informed consent was obtained from all participants or their legal guardians. All methods were performed in accordance with guidelines and regulations from our institutions’ IRB.

### Assessment of naming

Participants were tested using the Western Aphasia Battery-Revised (WAB-R)^20^ to screen different language abilities and obtain a global measure of aphasia. The Pyramids and Palm Trees Test (PPT)^21^ was used to determine semantic knowledge, and the Philadelphia Naming Test (PNT)^22^ was used to test confrontational naming. The PNT was repeated within one week to determine intra-subject variability. During the PNT, participants had 10 seconds to produce a response. The last complete attempt was used for scoring. Semantic paraphasias were defined as all incorrect real word responses related to the target in meaning. Phonological paraphasias were defined as real word attempts with phonological similarities as well as non-word attempts with incorrect phonemes that preserved more than 50% of the target word. Circumlocutions were scored as no response; dysfluency as articulation errors, and partially phonologically related words were considered to be phonological errors (or, if it was more than 50% different than the target, a neologism). In the case of visual errors (e.g., person says “house” for the “garage”), we redirected the subjects and clarified so they knew which part of the picture to respond to. If the visual errors persisted they were coded as semantic paraphasias. Mixed paraphasias and articulation errors were not included in the analysis. The PNT results from both sessions were averaged, and semantic and phonemic paraphasias were expressed as a percentage of naming attempts excluding the number of no responses since we were interested in semantic and phonemic paraphasias when naming was attempted. All standardized speech and language tests were administered and scored by an experienced American Speech-Language-Hearing Association-certified speech-language pathologist.

### Image acquisition

All participants were scanned on a Siemens 3 T TIM Trio MRI scanner (Siemens Healthcare, Erlangen, Germany) using a 12-channel head coil at the Medical University of South Carolina. Data were acquired in three series, with three degrees of diffusion weighting (b-value = 0, 1000, 2000 s/mm^2^), using 30 diffusion-encoding directions acquired twice for each b = 1000 and b = 2000, as well as eleven additional images without diffusion weighting (b = 0), for a total of 131 volumes. Additional acquisition parameters were: TE = 101 ms, TR = 6100 ms, 2.7 × 2.7 × 2.7 mm^3^ isotropic voxels, pixel bandwidth = 1355 Hz/px. All diffusion-weighted images (DWI) were acquired using a twice-refocused gradient scheme to minimize the contributions of eddy currents and without partial Fourier encoding. High resolution 1 mm^3^ isotropic T2-weighted images were acquired for lesion demarcation utilizing a 3D-TSE SPACE protocol (Field of view (FOV) = 256 × 256 mm^2^, 160 sagittal slices, TR = 3200 ms, TE = 212 ms, turbo factor = 129, echo trains per slice = 2, echo train duration = 432 ms) and, for anatomical reference, T1-weighted images were gathered using an isotropic 1 mm MPRAGE sequence: FOV = 256 × 256 mm, 9° flip angle, TI = 925 ms, TR = 2250 ms, TE = 4.15 ms.

### Image data processing

**Figure 1:**
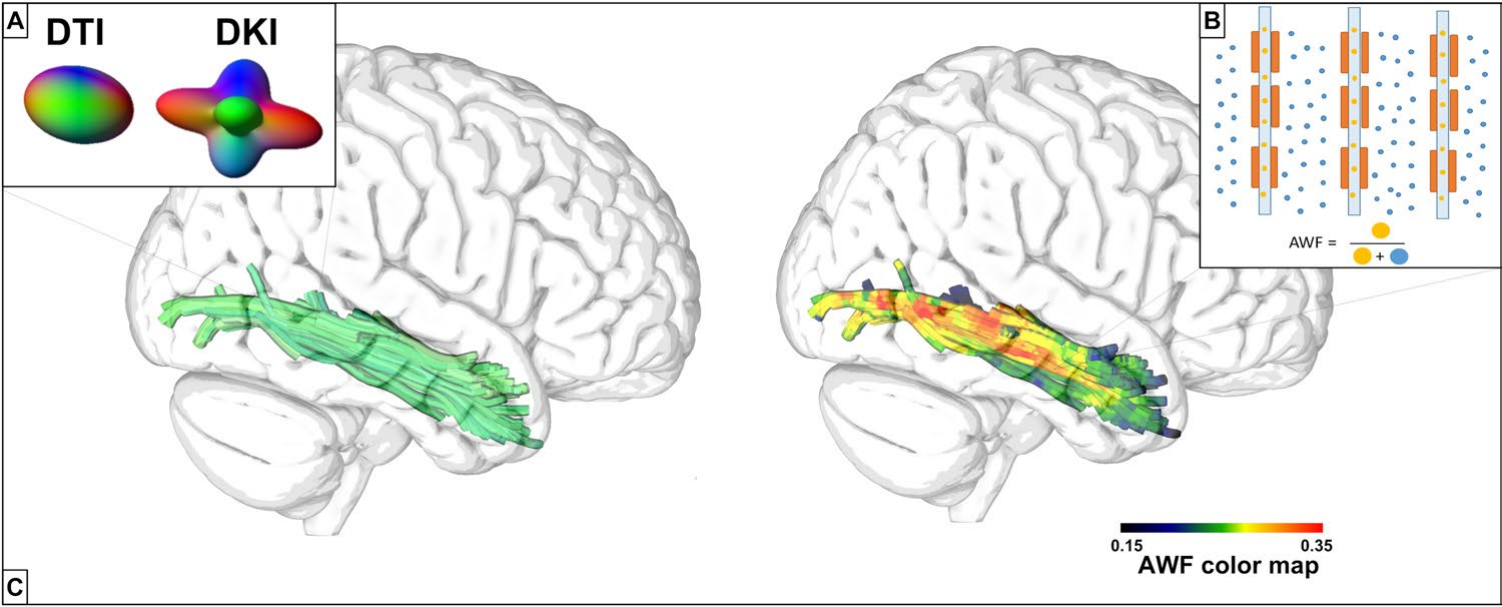
(**A**) DKI allows for voxel-wise estimation of the number of fiber directions through the calculation of a kurtosis diffusion orientation distribution function (dODF), while the dODF estimated from DTI provides only a single direction. (**B**) The AWF is the voxelwise ratio of intra-axonal water (orange) to the total water content (orange + blue). (**C**) (Left) The ILF estimated using kurtosis-based deterministic tractography with streamlines color-coded according to directionality. (Right) The ILF color-coded according to the underlying AWF values. Augmenting tractography with microstructural information paints a more complete picture of the underlying environment.

The assessment of tract-specific microstructure included the calculation of scalar diffusion metrics along the length of the white matter fibers. The terms *fiber* or *bundle* are used here to indicate the deterministic paths identified by diffusion tractography, which are the biophysical representation of large collections of axonal projections in white matter. In this study, we employed three novel forms of structural white matter analyses: (a) patient specific white matter tractography was performed using DKI, a technique that requires a multi-shell diffusion acquisition and dedicated post-processing procedures to improve upon tractography through the delineation of fiber crossings^23,24^; (b) the integrity of individual fibers was described using microstructural modeling metrics (i.e. AWF) derived from DKI^15^, which provide a more specific description of the underlying microstructure than conventional dMRI metrics (i.e. FA); and (c) AWF and FA- were assessed in a fine grained pattern along specific tracts, enabling a detailed assessment of where and how post-stroke integrity can affect language. Figure 1 highlights these methodologies; all techniques are explained in further detail below.

#### DKI tractography

DKI is an extension of the more conventional diffusion tensor imaging (DTI) method^25^ that provides a more thorough characterization of white matter microstructure, as well as more accurate fiber tractography^16,23^. In addition to the standard diffusion measures available with DTI, DKI also estimates the diffusional kurtosis, which quantifies the non-Gaussianity of the underlying water diffusion process^16^. The dMRI signal model for DTI can be expressed as

1 $$\mathrm{ln}\,S(b)-\,\mathrm{ln}({S}_{0})\approx -\,bD,$$

while the signal model for DKI is

2 $$\mathrm{ln}\,S(b)-\,\mathrm{ln}({S}_{0})\approx -\,bD+\frac{1}{6}{b}^{2}{D}^{2}K,$$

where $\,S(b)$ is the measured dMRI signal at diffusion weighting b,$\,{S}_{o}$ is the signal intensity for b = 0 s/mm^2^, $D$ is the apparent diffusion coefficient, and $K$ is the apparent diffusional kurtosis.

Analogous to the diffusion tensor, a kurtosis tensor, which describes the kurtosis’ dependence on direction, can be constructed with DKI^16^. The kurtosis tensor permits the assessment of the underlying microstructure through scalar metrics of kurtosis, which characterize the complexity of the brain cytoarchitecture, and it provides a voxel-wise description of fiber orientations and their crossings through the calculation of a kurtosis diffusion orientation distribution function (dODF) (Fig. 1)^26^. This is in contrast to other commonly used methods that more explicitly model fiber crossings (e.g. bedpostX)^27^. Previous work has demonstrated high sensitivity to pathology when using mean kurtosis as a measure of tissue microstructure^28–33^, as well as tractography that identifies intra-voxel fiber crossings that are not apparent with DTI^24^.

Diffusion and kurtosis tensors were estimated using publicly available post-processing software known as Diffusion Kurtosis Estimator^34^ (DKE, Medical University of South Carolina, Charleston, USA, https://www.nitrc.org/projects/dke/). To improve the signal-to-noise ratio, raw dMRI images were first denoised using a principal components analysis approach^35^, and, Gibbs ringing artifacts were removed using the method of Kellner *et al*.^36,37^. All DWI acquisitions including the additional images with b = 0 were linearly coregistered between themselves using FSL (FMRIB Software Library v5.0)^38^ before averaging them into a final set of 61 image volumes which was used as the input for DKE.

To localize ventral and dorsal stream white matter, we constructed tractography seed masks for two major components of the dual stream pathway: Inferior Longitudinal Fasciculus (ILF) (ventral stream) and Superior Longitudinal Fasciculus (SLF) (dorsal stream). For each participant, we located the core of each white matter bundle by calculating the intersection between the JHU atlas^39^ (SLF thresholded at 21% (20/97) and ILF at 25% (20/79)) and a white matter probability mask (thresholded at 50%) created using SPM12, while excluding any lesioned voxels that were drawn on the T2-weighted images. Lesion drawings included the entire post-stroke cavity as well as areas with laminar necrosis and gray and white matter gliosis. Enantiomorphic unified segmentation-normalization^40^ was employed to calculate the spatial transformation between MNI and native T1-space (Clinical Toolbox, SPM12^41^) taking into account lesions. This transformation was used to transfer the JHU atlas to native T1-weighted space, while the linear transformation from native T1-weighted to native diffusion space was calculated using FSL (FMRIB Software Library v5.0).

The individual ILF and SLF seed masks were used for deterministic kurtosis-based white matter tractography using the FT-toolbox from DKE, which estimates fiber directions from both diffusion and kurtosis tensors. Streamlines were removed when the probability of belonging to a neighboring white matter bundle, defined by the overlap between the streamline coordinates and the JHU ROIs, was larger than the probability of belonging to the target bundle. Additional tractography parameters were FA-threshold = 0.1, angular threshold = 35°, minimum track length = 20 mm, step size = 1 mm and seed number = 1000.

White matter lesion overlap was calculated as the cross-section between the manually delineated lesion mask and the third quartile of the JHU SLF and ILF ROIs. The third quartile was chosen to provide an inclusive representation of white matter lesion overlap, i.e., regions of the tract that were damaged. Crucially, all analyses were performed in native diffusion space, reducing interpolation artifacts.

#### Microstructural modeling: Axonal Water Fraction

From the kurtosis tensor, it is also possible to calculate information related to specific microstructural compartments (i.e., axonal vs. extra-axonal)^15^. AWF is a metric, obtained through the white matter tract integrity (WMTI) method, which estimates the relative amount of water inside axons to the total water content within one voxel (Fig. 1)^15^. Specifically, AWF is estimated by

3 $$AWF=\frac{{K}_{max}}{{K}_{max}+3},$$

where ${K}_{max}$ is the maximal directional kurtosis calculated from the kurtosis tensor over all possible directions. Tissue modelling leverages neurobiological characteristics from tissue structure, which are translated into biophysical properties as follows: (1) axons can be approximated to long impermeable thin cylinders that are mostly coplanar, (2) diffusion in the extra-axonal water pool can be approximated as Gaussian, and 3) water exchange between compartments is negligible during the MRI sampled diffusion time (~50 ms). Except for the coplanarity condition, these are generally accepted properties of white matter that have been assumed in a variety of different tissue modeling approaches^42^. Because the model requires the axons to be coplanar, Eq. [3] may not be accurate in some white matter regions with complex patterns of fiber crossings^15^.

In summary, we focused on the conventional metric FA, calculated from the diffusion tensor, and the tissue modeling metric AWF, determined from the kurtosis tensor (Eq. [3]). AWF was calculated from the kurtosis tensor using in-house scripts. FA was calculated using a DKI-based approach to improve its accuracy^43^.

#### Along tract metrics

**Figure 2:**
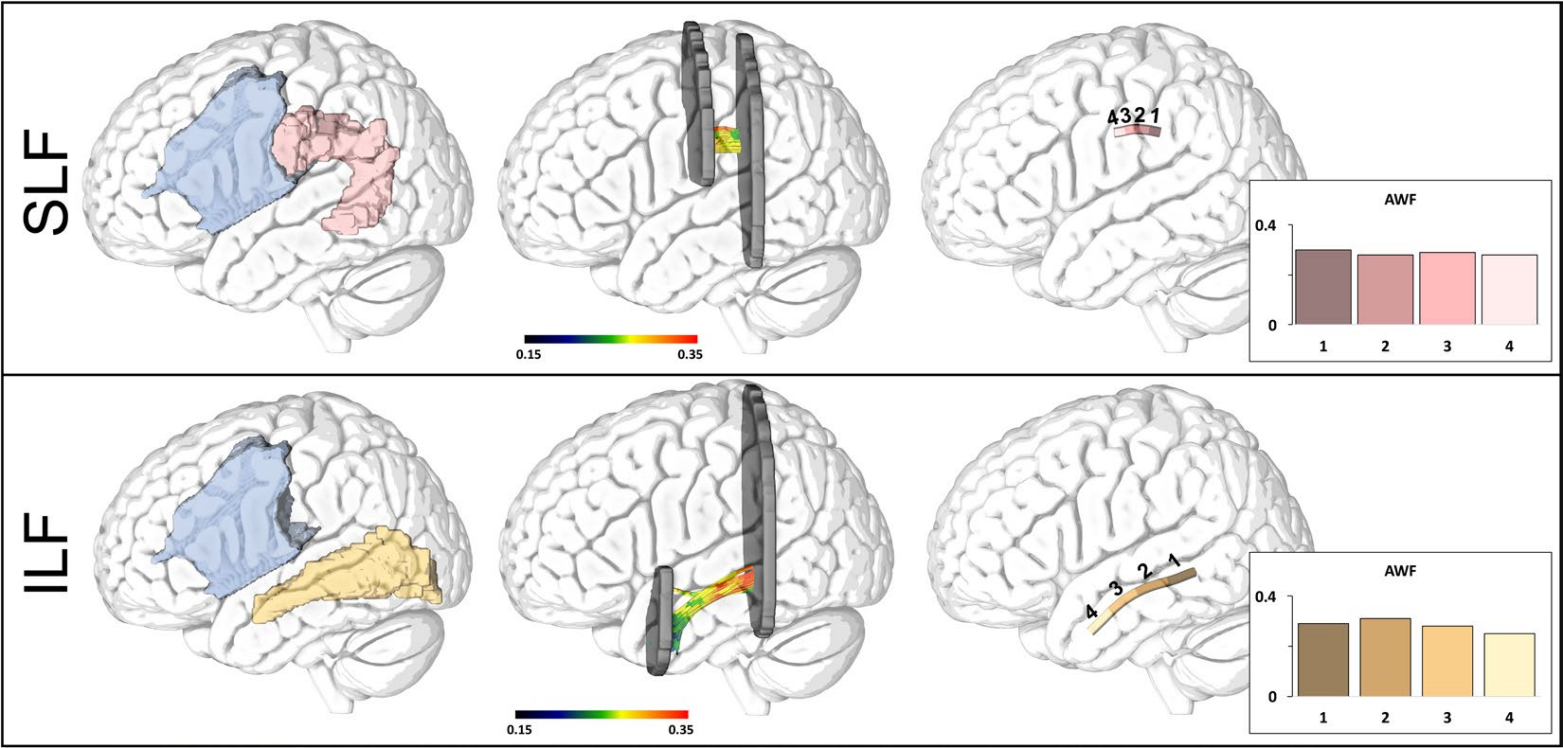
Image processing pipeline used to construct the average SLF and ILF for a representative individual. Individual seedmasks (left) for the ILF (beige) and the SLF (pink) were constructed and used as starting points for deterministic tractography. Lesioned voxels (blue) were excluded from each seedmask. The tractography results were cropped (middle), averaged and divided into 4 different segments (right) in each of which we calculated the average AWF.

Figure 2 demonstrates how FA and AWF were quantified along the length of two distinct white matter fiber bundles. To enable comparisons between individuals, we isolated the core of each bundle using a methodology based in part on AFQ^44^. While the AFQ pipeline performs whole brain seeding and uses the JHU atlas to assign each streamline to their respective fiber bundle, we created different seed masks to locate the specific bundles. The isolation of the core was performed based on the methods proposed in the original AFQ description with the exception that a straightforward averaging was used to identify the final values compared to a distance-weighted average in AFQ. Specifically, each subject’s fiber bundle was cropped at similar locations employing the JHU-ROIs^45^, with each streamline being interpolated into 100 equidistant points using cubic B-splines^46^ (Fig. 2, middle). Cropping provides the streamlines with similar starting and ending points, which facilitates the identification of the core components of each tract. By assessing equivalent segments across patients, it is possible to relate their integrity with individualized naming performance through statistical analyses. AWF and FA values were quantified at each point, but to facilitate statistical analysis, we assigned the average metric to each point along the fiber bundle’s geometric mean resulting in 100 values along the length of the ILF and SLF sections that were ultimately binned into 4 segments containing 25 nodes each (Fig. 2, right). In what follows, these midsections will be referred to as the SLF and ILF.

#### Gray matter necrosis

To investigate the interaction between dual stream gray matter necrosis and white matter integrity, we defined three gray matter ROIs: supra-Sylvian language-processing gray matter regions (dorsal stream) infra-Sylvian language-processing gray matter regions (ventral stream) identified using fMRI^47^ and the SMG. Gray matter necrosis was calculated as the percent overlap between the stroke lesion and the gray matter ROIs as defined by the JHU atlas^48^. Suprasylvian gray matter included the left posterior middle frontal gyrus (pMFG), the left inferior frontal gyrus (IFG) pars opercularis, the left IFG pars triangular, and the left angular gyrus (AG). Infrasylvian gray matter was composed by the superior temporal gyrus (STG), the pole of the STG, the middle temporal gyrus (MTG), the posterior superior temporal gyrus (pSTG) and the posterior middle temporal gyrus (pMTG). Damage to the SMG was also quantified since it has been implicated in phonological processing^8^. Lesion locations can be found in Supplementary Fig. 1.

### Statistical analysis

We created multiple linear regression models to assess the relationship between paraphasias and integrity of the ventral and the dorsal streams. Specifically, we used the percentage of semantic or phonemic paraphasias as dependent variables in two separate multiple linear regression models with SLF integrity (i.e., AWF and FA), as well as ILF integrity at four distinct tract locations as predictors controlling for white matter lesion overlap. We also assessed whether the inclusion of gray matter damage influenced the model. All reported correlation coefficients are Pearson *r*. *P*-values were adjusted using the Bonferroni correction. Fisher r-to-z transformations were performed to investigate if the dissociations between the dorsal and the ventral stream integrity and semantic and phonemic paraphasias were statistically significant.

## Results

### Language tests

WAB aphasia quotient (WAB-AQ), aphasia types and average Philadelphia Naming Test results for all individuals are summarized in Supplementary Table S1. Aphasia ranged from severe to mild (WAB-AQ = [20.1–93.7]), with an average (±standard deviation) WAB-AQ of 54.1 (±22.5). During confrontational naming, participants elicited an average of 36.0% (±27.1) correct responses. Incorrect responses were identified as semantic paraphasias 14.7% (±12.5) and phonemic paraphasias 7.1% (±8.3) of the time. The Pyramids and Palm Trees Test [20] demonstrated that the semantic association pathway between pictures and their meaning was relatively preserved in the majority of subjects (45 ± 5).

### Tract-Based Integrity Analysis

**Figure 3:**
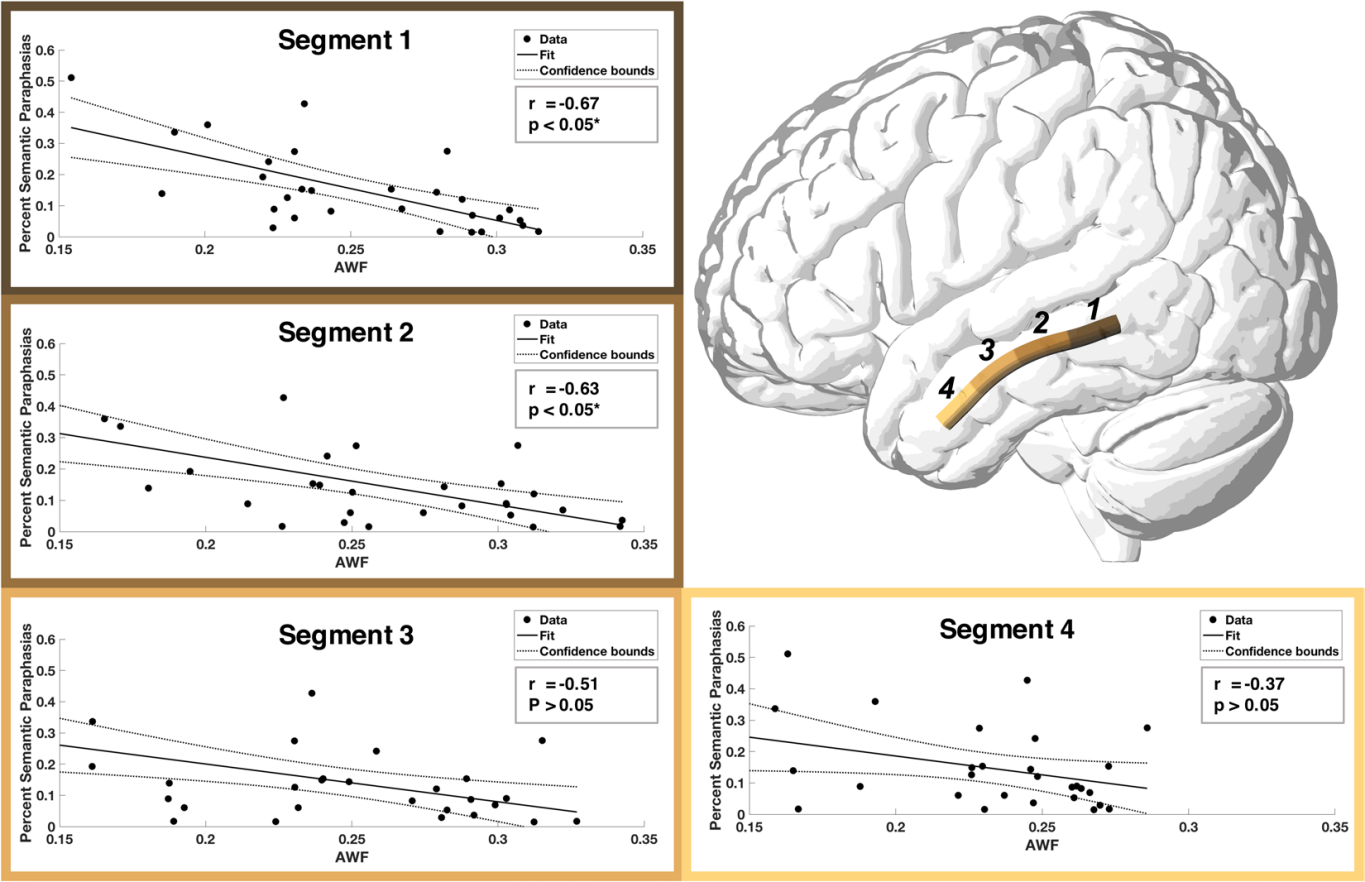
Scatterplots demonstrating the relationship between AWF and percent semantic paraphasias. Average AWF was calculated for different segments of equal size along the length of the ILF. A significant association was seen between the AWF and semantic paraphasias in the two most posterior segments (I & II; r = −0.67, r = −0.63) (after correction for multiple comparisons).

As explained in the methods, we used along tract quantification to assess AWF and FA along the longitudinal length of individual fiber bundles in order to provide a more detailed evaluation of tract integrity. Both the residual ILF and SLF were identified in 18 out of 32 subjects, while only the residual ILF was located in an additional 11 subjects. There were no cases where the SLF was not accompanied by the ILF. All subsequent tract-based analyses were performed on these residual connections. Tables 1 and 2 summarize the interactions between 1) the percentage of semantic and phonemic paraphasias and 2) the average AWF (Table 1) and FA (Table 2) at different parts along the ILF and SLF. As described in Table 1, semantic paraphasias had the highest relationship with the average AWF within the posterior portion of the ILF (segment 1: r = −0.67 and segment 2: r = −0.63, p < 0.05 corrected). Figure 3 shows the scatter plots (and their line of best fit with 95% confidence intervals) demonstrating the distribution of the percentage of semantic paraphasias and the AWF from each segment of the ILF. The FA of sections 1, 2 and 3 were also significantly correlated with semantic paraphasias (r = −0.66, r = −0.56 and r = −0.58, p < 0.05 corrected). However, neither AWF nor FA along the length of the SLF was associated with the percentage of semantic paraphasias.

**Table 1:** Summary of all correlation coefficients between average AWF, calculated along the length of the ILF and the SLF, and both phonemic and semantic paraphasias.

Dependent Variable	Region	Segment	r	r (corrected for lesion overlap)
Semantic Paraphasias	ILF	I	−0.67*	−0.49*
		II	−0.63*	−0.40
		III	−0.51	—
		IV	−0.36	—
	SLF	I	0.21	—
		II	0.35	—
		III	0.42	—
		IV	0.31	—
Phonemic Paraphasias	ILF	I	0.25	—
		II	0.14	—
		III	0.15	—
		IV	0.08	—
	SLF	I	−0.68*	−0.65*
		II	−0.52	—
		III	−0.47	—
		IV	−0.39	—

(*p < 0.05, corrected for multiple comparisons).

**Table 2:** Summary of all correlation coefficients between FA, calculated along the length of the ILF and the SLF, and both phonemic and semantic paraphasias.

Dependent Variable	Region	Segment	r	r (corrected for lesion overlap)
Semantic Paraphasias	ILF	I	−0.66*	−0.44
		II	−0.56*	−0.31
		III	−0.58*	−0.37
		IV	−0.3	—
	SLF	I	0.07	—
		II	0.16	—
		III	0.25	—
		IV	0.06	—
Phonemic Paraphasias	ILF	I	0.12	—
		II	0.13	—
		III	0.15	—
		IV	0.16	—
	SLF	I	−0.46	—
		II	−0.29	—
		III	−0.24	—
		IV	−0.33	—

(*p < 0.05, corrected for multiple comparisons).

The weak association between semantic paraphasias and the SLF diffusion measurements was not related to the smaller sample size of SLF compared to ILF (18 vs. 29). For a similar r-value (0.67), the statistical power from n = 29 to n = 18 drops from 0.98 to 0.88. We performed 1000 random sampling of the ILF with n = 18, the average r-value was 0.65, and r = 0.21 was below the 99^th^ percentile of this sample. For this reason, the relationship between the SLF and semantic paraphasias was not significant taking into account the smaller sample size. In addition, Fisher r-to-z demonstrated that the AWF within the ILF (segment 1) related more strongly to semantic paraphasias than the AWF within the SLF (segment 1) (p < 0.05).

**Figure 4:**
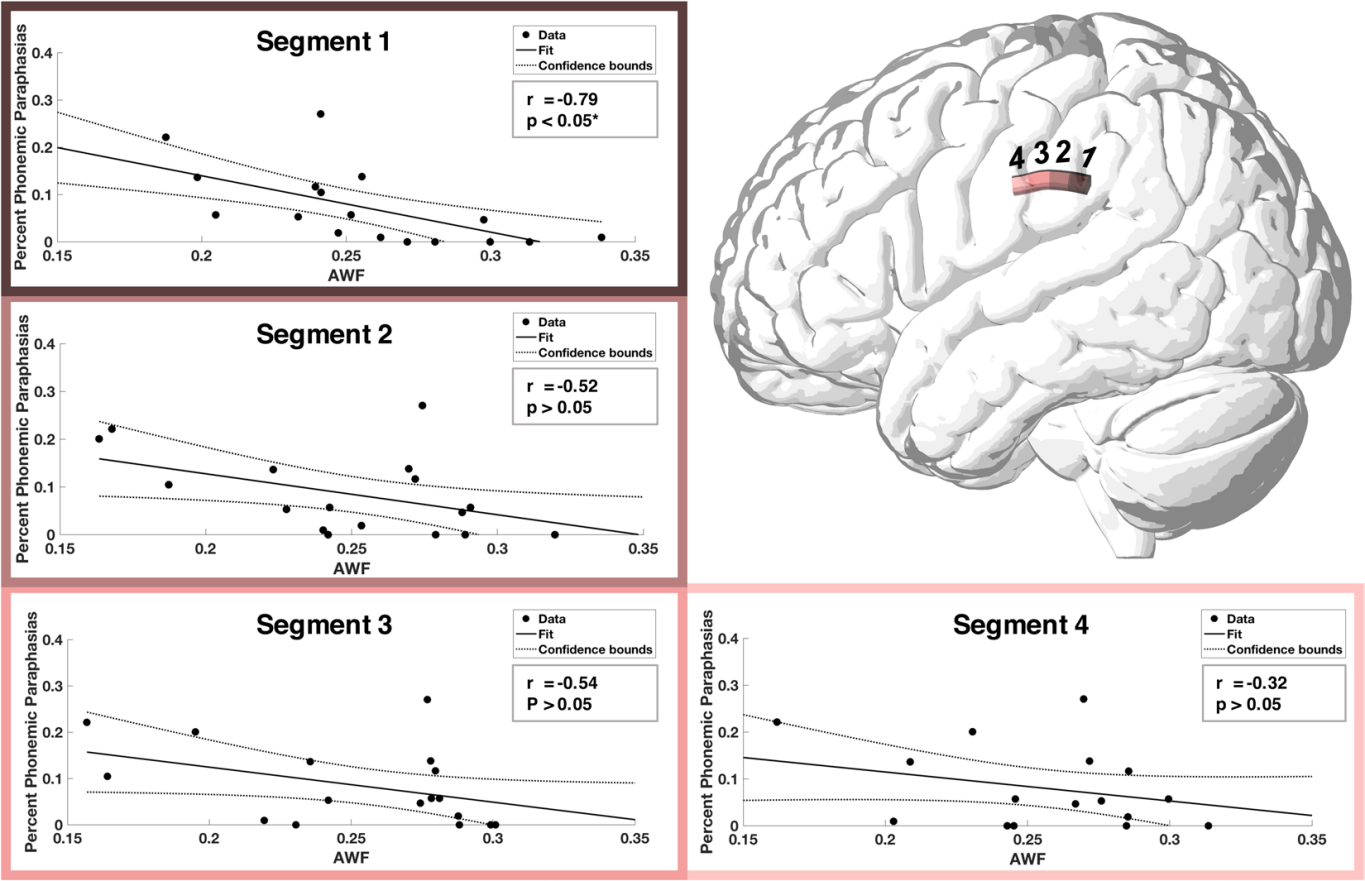
Scatterplots demonstrating the relationship between AWF and percent phonemic paraphasias. Average AWF was calculated for different segments along the length of the SLF. A significant association was seen between the AWF and phonemic paraphasias in the two most posterior segments (I; r = −0.68) (after correction for multiple comparisons).

Phonemic paraphasias had the most significant association with the average AWF within the most posterior part of the SLF (segment 1: r = −0.68, p < 0.05) (Table 1). The distribution of phonemic paraphasia prevalence and average AWF calculated from the different parts of the SLF is depicted in Fig. 4. The FA of segment 1 was weakly associated with phonemic paraphasias, albeit not surviving correction for multiple comparisons (r = −0.46, p > 0.05). The percentage of phonemic paraphasias was not related to any of the diffusion metrics calculated from the ILF. Fisher r-to-z transformations confirmed that phonemic paraphasias were more associated with AWF within segment 1 of the SLF than within segment 1 of the ILF (p < 0.05).

It is important to highlight that ILF lesion burden was associated with the percentage of semantic paraphasias (r = 0.56, p < 0.05). Therefore, to exclude this potential confounder from the analyses relating paraphasias with tract-based microstructure, we recalculated the linear models described above (whose r and p values mentioned above did not control for lesion burden) now controlling for lesion overlap with the ILF and SLF. This stricter analysis resulted in two remaining significant correlations between 1) the average AWF in segment 1 of the ILF and the percentage of semantic paraphasias (r = −0.49, p < 0.05) and 2) the average AWF in segment 1 of the SLF and the percentage of phonemic paraphasias (r = −0.65; p < 0.05). AWF and FA were strongly correlated (ILF: r = 0.81, p < 0.05; SLF: r = 0.74, p < 0.05), but stepwise regression (based on an F-test of the change in the sum of squared error by adding a term) using AWF, FA, and lesion overlap as input variables resulted in a final linear model with AWF as the only variable.

### Impact of gray matter necrosis

The degree of infra-Sylvian gray matter necrosis was associated with the frequency of semantic paraphasias made during confrontational naming (r = 0.42, p < 0.05). However, when correcting gray matter damage for the average AWF of the posterior ILF (segment 1), the relationship did not exceed statistical significance. Conversely, a statistically significant relationship between the AWF of the posterior ILF and semantic paraphasias (r = −0.67, p < 0.05) remained when adjusting for infra-Sylvian gray matter necrosis (r = −0.55 p < 0.05). Supra-Sylvian specific gray matter was not related to percent phonemic paraphasias and thus did not alter the relationship between the AWF of the SLF and phonemic paraphasias. Note that the AWF calculated from SLF’s segment 1 was associated with supra-marginal gyrus (SMG) necrosis (−0.55, p < 0.05); however, SMG necrosis only weakly related to the percentage of phonemic paraphasias (r = 0.31, p = 0.21).

## Discussion

In this study, we examined the importance of residual white matter pathways supporting semantic and phonological processing during confrontation naming in individuals with chronic post-stroke aphasia. We used advanced dMRI post-processing techniques to trace representative large pathways in the ventral and dorsal streams (ILF and SLF, respectively) and to measure a microstructural property of axonal integrity (AWF) along the fibers in each tract. Our results indicated a double dissociation between white matter axonal loss and semantic vs. phonemic naming impairments: AWF in the ILF (particularly in the posterior aspect of the tract) was associated with the number of semantic paraphasias, but not with the number of phonemic paraphasias. Conversely, AWF in the SLF (also more strongly in the posterior aspect of the tract) was related to the number of phonemic paraphasias, but not with the number of semantic paraphasias.

These findings are in line with the dual stream model of language, which proposes the presence of functionally and anatomically distinct processing routes for lexical access (i.e., ventral stream) and phonological form encoding (i.e., dorsal stream). Importantly, they provide anatomical confirmation at the white matter network level and complement existing lesion based studies, which mostly focus on regional damage, not residual white matter connections. Moreover, these results also provide information on the relevance of the biological nature of damage, i.e., axonal loss, and their location within the white matter tracts.

**Figure 5:**
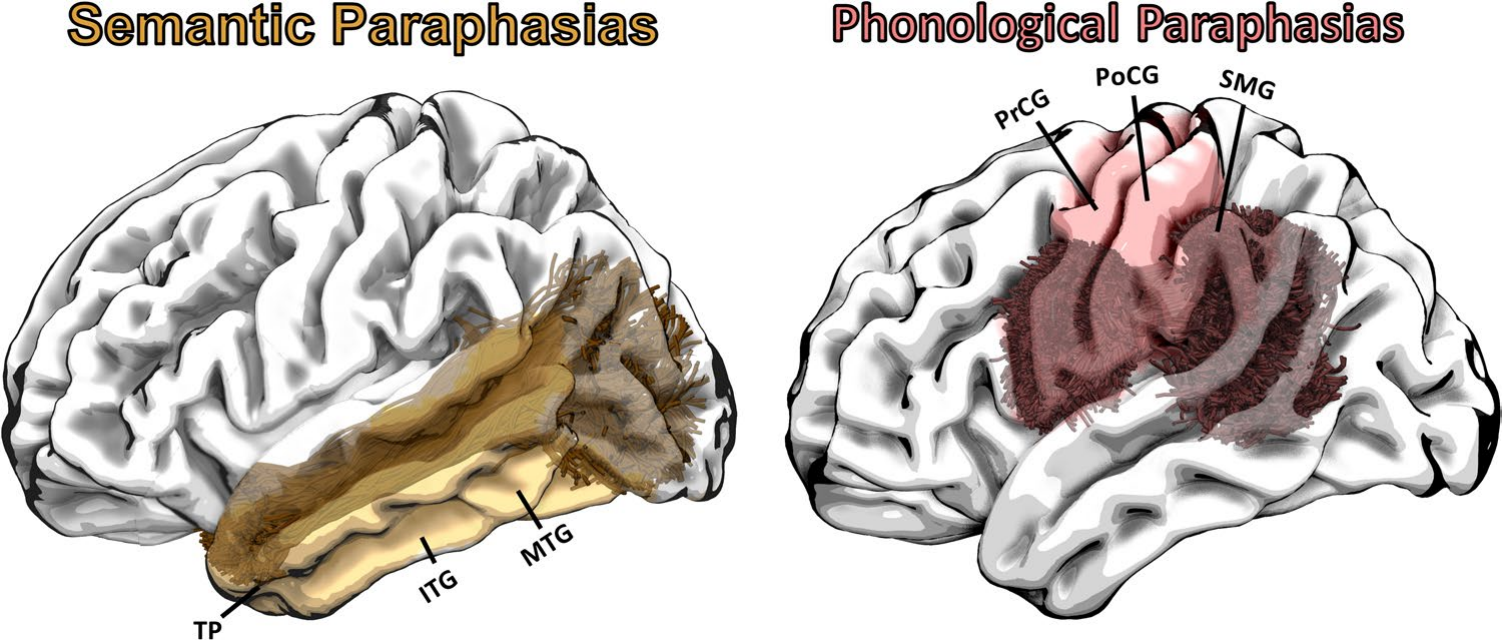
Left: Semantic paraphasias have been associated with damage to a multitude of temporal gray matter regions (e.g., TP, STG, ITG, and MTG)^52,8,9,11,49,56^. Here, we demonstrated that axon density of the ILF relates to the frequency of semantic paraphasias in individuals with post-stroke aphasia. The ILF interconnects these gray matter regions^51^ likely supporting parts of a semantic network^12^. Right: Damage to the PrCG, PoCG and the SMG has been shown to result in phonemic paraphasias^7,8,12,13^. These areas are interconnected primarily through the SLF and arcuate fasciculi, which bridge perisylvian frontal, parietal, and temporal cortices. A greater degree of axonal loss in the posterior SLF related to a larger number of phonemic paraphasias. TP = Temporal Pole, STG = Superior Temporal Gyrus, ITG = Inferior Temporal Gyrus, MTG = Middle Temporal Gyrus, PrCG = Precentral Gyrus, PoCG = Postcentral Gyrus, SMG = Supramarginal Gyrus.

Multiple studies have demonstrated that regional post-stroke brain damage can be associated with naming errors, with damage to dorsal regions relating mostly to phonemic paraphasias, and damage to ventral regions predominantly associated with semantic paraphasias. Figure 5 demonstrates the positioning of the ILF and SLF relative to the gray matter regions most often implicated in naming impairments. Different studies have reported that semantic errors are related to lesion involvement of the temporal pole^7,9^, the inferior temporal gyrus^49^, as well as different portions (anterior, mid and posterior) of the MTG^7,8,10,11,50^. Taken together, it is possible that the intactness of these areas, in combination with the successful integration within the language network is required for unimpaired lexical access. The association between neuronal damage and word production errors has recently been further explored in relation to the Dell speech production model^50^. It is important to note that these studies did not focus on the residual integrity of the white matter as quantified by diffusion MRI, but instead focused on lesion location using structural MRI. We believe that our results complement their conclusions highlighting the importance of residual white matter integrity in concordance with cortical and white matter damage as shown on structural MRI.

The ILF, whose fibers link the superior- middle- and inferior temporal gyri^51^, has been suggested by task-based functional MRI studies to support such a semantic network^12,52^. Phonology and articulatory representations are supported by the dorsal stream, and damage to the SMG and post-central gyrus has especially been related most frequently to impaired phonological encoding^7,8,12,13^. Schwartz *et al*. postulated in a structural voxel-based lesion-symptom mapping analysis that phonological paraphasias might also arise from damage to dorsal stream white matter pathways^8^. Here, we directly located the residual white matter pathways using dMRI, and demonstrated that the axon density of the residual SLF indeed relates to the frequency of phonemic paraphasias, confirming the hypothesis by Schwartz *et al*. In addition, we also demonstrated that the degree of axonal loss of the residual ILF relates with semantic paraphasias during confrontational naming, independent of the degree of damage to the MTG. Our results provide evidence that damage to the gray matter regions as well as the integrity of the remaining connections between them should be considered when studying naming impairments after stroke. More specifically, damage to the brain at the white matter network level can directly influence ventral and dorsal stream processing and lead to speech production errors. In comparison with the existing literature, this is the main novel finding from this study.

It is important to note that the diffusion imaging methods used in this study are relatively different from conventional fiber tracing methods^53^. We employed an approach to best define the integrity of the residual white matter networks in stroke survivors and elucidate the nature and location of the white matter post-stroke damage. The specific innovations of the methods used here are threefold: 1) the use of DKI to track white matter fibers and ameliorate tracing inaccuracies in areas of fiber crossing or complex curvature; 2) the use of DKI tissue modeling (i.e., WMTI) to approximate axonal loss (AWF); and 3) the quantification of microstructural tissue properties along the main axis of each tract. This allows for the direct assessment of the local integrity of specific tracts since it entails the actual tracing of each tract in each individual. Since tracts can follow unusual paths in lesioned brains, scalar diffusion metrics obtained from ROI or skeletonized based analyses, without tracing connections, could miss important individual details.

Studying changes along the main axis of the fibers was pioneered through a technique known as automated fiber quantification (AFQ)^44^, which is particularly useful when disease affects different parts of a fiber bundle to a different degree or when different segments of white matter pathways are composed of branches to different parts of gray matter. In a recent study, we used AFQ and demonstrated that aphasia recovery mediated plasticity varies along the length of a specific tract^33^. Here, we used the same principles of AFQ to determine AWF along the tracts, and we observed that damage to the posterior parts of the ILF and the SLF was more significantly associated with paraphasias highlighting the importance of local white matter integrity. While the explanation for these observations remains unclear, it may be related to the regional integration performed by these parts of the ILF, and SLF, with their posterior relationship to somatosensory processing areas in the temporal-parietal region, such as area Spt^54^. Alternatively, information transmission could preferably flow posteriorly to anteriorly where damage to segment 1 would impact the functioning of the downstream segments 2, 3, and 4. Lastly, it could be due to the posterior parts being more commonly lesioned after middle cerebral artery strokes and thereby strengthening the statistical association between damage and paraphasias in those areas. From our findings, we can conclude that posterior damage to either stream is a strong marker of paraphasias, but a more detailed dissection focusing on the regions connected by the specific streams is necessary.

It is a limitation of this study that only the ILF and the SLF were studied in a small number of cases without the assessment of other tracts and other naming errors related to the multiple subnetworks that form the dual stream system. It is important to emphasize that the nomenclature of white matter fibers is inconsistent throughout the literature (e.g., arcuate fasciculus vs. SLF). In this paper, the names ILF and SLF were chosen in accordance with the John-Hopkins University (JHU) atlas labeling convention used to seed the tracks. The SLF, however, likely includes fibers from both the SLF and the arcuate fasciculus, since no filtering algorithm based on fiber curvature was implemented. Similarly, the posterior location of the ILF also highly overlaps with the location of the IFOF. A thorough dissection of the tracks is however beyond the scope of this paper, and future work should focus on studying white matter connections between specified gray matter regions. Another caveat would be that the AWF is based on assumptions and simplifications about the properties of axons. For example, the WMTI model assumes that in a voxel all axons lie in the same plane, which might be more or less accurate in different locations^15^. In lesioned brains, extra-cellular and glial changes could add confounders, but these are likely less pronounced in chronic strokes, where the brain parenchyma is more stable and inflammatory responses and edema are minimal. Regardless, the metric will always represent a specific property of the underlying water diffusion processes, albeit not necessarily the axon density. Finally, a decrease in AWF could also be related to an increase in water in the extra-axonal compartment. However, that seems unlikely to occur in isolation without the loss of any axons. Note that the same error types have been associated with more than one cause and revealing a one to one relationship between brain damage and paraphasias would be unrealistic^55^. Future work should focus on determining the integrity of the entire dual stream system and on studying the relationship between its residual structure and naming performance. Studying additional error types and further subcategorizing semantic and phonemic paraphasias might provide additional information about the origin of naming errors in post-stroke aphasia^56,57^.

In summary, our results are in concordance with the dual stream model of language processing and further corroborates the notion that, during speech production, knowledge association (semantics) depends on the integrity of ventral, whereas form encoding (phonemics) is localized to dorsal pathways. These findings also demonstrate the importance of the local residual integrity of specific white matter pathways beyond regional gray matter damage for speech production and underscore how biophysical tissue models can yield more specific and interpretable results for clinical translation.

## Electronic supplementary material

Supplementary information

## Data Availability

Readers can access data by contacting L.B., who will provide a secure link to a downloadable folder including both de-identified neuroimaging and behavioral data.
