# Supplementary material for: Types of naming errors in chronic post-stroke aphasia are dissociated by dual stream axonal loss
Source: Sci Rep. 2018 Sep 25;8:14352. doi: 10.1038/s41598-018-32457-4 (PMC6156587; doi:10.1038/s41598-018-32457-4)
Supplement: Supplementary file 1 — Supplementary information [file 41598_2018_32457_MOESM1_ESM.pdf]

Types of naming errors in chronic post-stroke aphasia are dissociated by dual stream axonal loss

Emilie T. McKinnon[a,b,c], Julius Fridriksson[d], Alexandra Basilakos[d], Gregory Hickok[e], Argye E. Hillis[f], M.Vittoria Spampinato[g], Ezequiel Gleichgerrcht[a], Chris Rorden[h], Jens H. Jensen[b,c,g], Joseph A. Helpern[a,b,c,g], Leonardo Bonilha[a,b,c]

[a] Department of Neurology, Medical University of South Carolina, 96 Jonathan Lucas St, Charleston, SC 29425, USA.

[b] Center for Biomedical Imaging, Medical University of South Carolina, 96 Jonathan Lucas St, Charleston, SC 29425, USA.

[c] Department of Neuroscience, Medical University of South Carolina, 173 Ashley Avenue, Charleston, SC 29425, USA.

[d] Department of Communication Sciences and Disorders, University of South Carolina, 921 Assembly Street, Columbia, SC 29208, USA.

[e] Department of Cognitive Sciences, Center for Language Science and Center for Cognitive Neuroscience, University of California, 2201 Social & Behavioral Sciences Gateway Building, Irvine, CA 92697, USA

[f] Department of Neurology, Johns Hopkins University, 725 N Wolfe St, Baltimore, MD 21205, USA

[g] Department of Radiology and Radiological Science, Medical University of South Carolina, 96 Jonathan Lucas St, Charleston, SC 29425, USA.

[h] Department of Psychology, University of South Carolina, 1512 Pendelton Street, Columbia, SC 29208, USA.

Running title: Dual stream axonal loss and naming in aphasia

Address correspondence to:
Leonardo Bonilha, M.D., Ph.D.
Department of Neurology
Medical University of South Carolina
96 Jonathan Lucas St.
Charleston, SC, Zip: 29425
Bonilha@musc.edu
+1/843.792.5044

Supplementary Information

Table S1: Subject demographics and language battery results. (M=male; F=female)

Participant	Gender	Race	Age (years)	Time Post-Stroke (month)	WAB-AQ	Aphasia Type	Correct Responses (%)	Self Correct (%)	Semantic Paraphasias (%)	Phonemic Paraphasias (%)	Mixed Paraphasias (%)	No respones (%)
1	M	white	76	9	87.8	Anomic	72.4	10.49	12.01	0	0	5.11
2	F	white	66	13	80.3	Anomic	60.87	8.7	24.14	1.89	0	38.6
3	M	white	70	101	62.7	Broca's	34.94	11.7	3.61	0.3	0	5.43
4	M	white	68	48	87.2	Broca's	70.69	12.7	15.3	0.29	0	6.02
5	M	white	38	20	41.8	Broca's	42.5	5.8	27.55	13.76	0	45.87
6	M	white	66	47	77.8	Anomic	47.81	4.7	1.46	0	0	2.08
7	M	black	50	78	80.8	Broca's	44.3	4.7	6.09	22.08	0	10.83
8	M	white	49	6	20.1	Broca's	26.39	5.6	15.3	27.04	0	143.1
9	M	black	42	108	31.2	Global	36.36	1.8	51.11	3.07	0	234.09
10	F	white	37	21	52.8	Broca's	66.8	5	19.69	3.85	0.38	35.19
11	M	white	69	9	59.9	Broca's	25.1	17.8	12.6	11.64	2.95	36.85
12	M	black	64	6	45.2	Conduction	1.75	6.4	5.28	5.25	0	2.35
13	M	black	58	25	93.7	Anomic	77.08	10.1	8.63	0	0.3	4.17
14	M	black	47	25	27.9	Broca's	7.69	1.1	11.32	14.74	2.08	395.18
15	F	white	64	86	42.9	Wernicke's	8.87	14.8	14.86	10.45	1.08	88.32
16	M	white	49	15	29.3	Broca's	0.8	0.8	1.68	5.69	1.02	193.7
17	M	white	55	84	23.4	Global	3.57	1.4	6.02	1.04	0	27.35
18	F	Asian	46	27	39.6	Broca's	29.96	7.7	19.24	13.6	2.19	42.54
19	F	white	48	21	32.3	Broca's	0.31	0.3	1.57	0.95	0.33	8.96
20	M	white	60	26	27.8	Broca's	0.65	2.3	8.97	20.07	1.1	15.81
21	M	white	55	9	67.9	Conduction	35.84	13.6	6.95	24.3	3.3	5.44
22	M	black	51	72	41.6	Broca's	17.17	6.3	35.94	8.33	3.31	5.73
23	M	white	62	60	86.8	Anomic	78.3	14.4	2.89	2.38	0.6	2.71
24	M	white	56	65	74	Conduction	79.1	6.9	8.73	0	0.6	4.64
25	M	white	64	8	50.9	Wernicke's	10.17	3.2	42.73	0.87	0.58	1.75
26	F	white	70	11	41.9	Wernicke's	7.26	6.4	27.41	0.92	0	50.28
27	M	white	74	7	60.2	Wernicke's	24.01	7.9	8.88	4.61	2.96	15.13
28	M	white	56	23	47.3	Broca’s	29.77	4	14.36	1.34	2.34	17.06
29	F	black	29	19	49.3	Broca’s	43.84	7.6	33.62	0.38	2.15	26.98
30	F	white	73	16	92.1	Anomic	85.02	4.9	8.25	0	0	7.03
31	M	white	50	23	30.1	Broca’s	60.16	2.4	13.86	5.67	2.45	184.57
32	M	white	61	22	43.3	Broca’s	23.43	3.8	1.65	21.34	2.11	46.57

Perseverations (%)	Unrelated (%)	Neologism (%)	Artic Errrors (%)
0	0.3	0	4.8
0.83	2.31	0	1.21
0	0.61	0	48.8
0	0.29	0	0.86
5.38	4.61	0.41	0
0	0	0	46.04
0	0	0	22.84
2.74	14.62	8.32	0
1.91	3.4	0	0
1.57	1.56	1.14	0
5.13	12.77	11.03	0.87
1.48	2.92	76.01	0.89
0	1.2	0.3	2.37
39.05	7.15	14.18	1.49
29.67	4.76	12.74	0
3.65	6.42	80.22	0
70.16	13.1	4.78	0
8.9	12.53	4.51	1.5
68.79	7.77	19.68	0.33
7.21	14.26	44.85	0.74
0	1.82	12.68	1.51
0	7.13	18.49	3.31
0	0.29	0.29	0.86
0	0.31	0.91	3.54
13.65	24.72	3.79	0.29
34.18	22.37	1.38	0
6.25	8.88	28.62	7.89
30.13	11.37	2.34	4.34
1.77	5.4	1.42	3.68
0	1.22	0	0.61
3.24	3.25	0	8.92
4.59	7.49	26.97	8.76

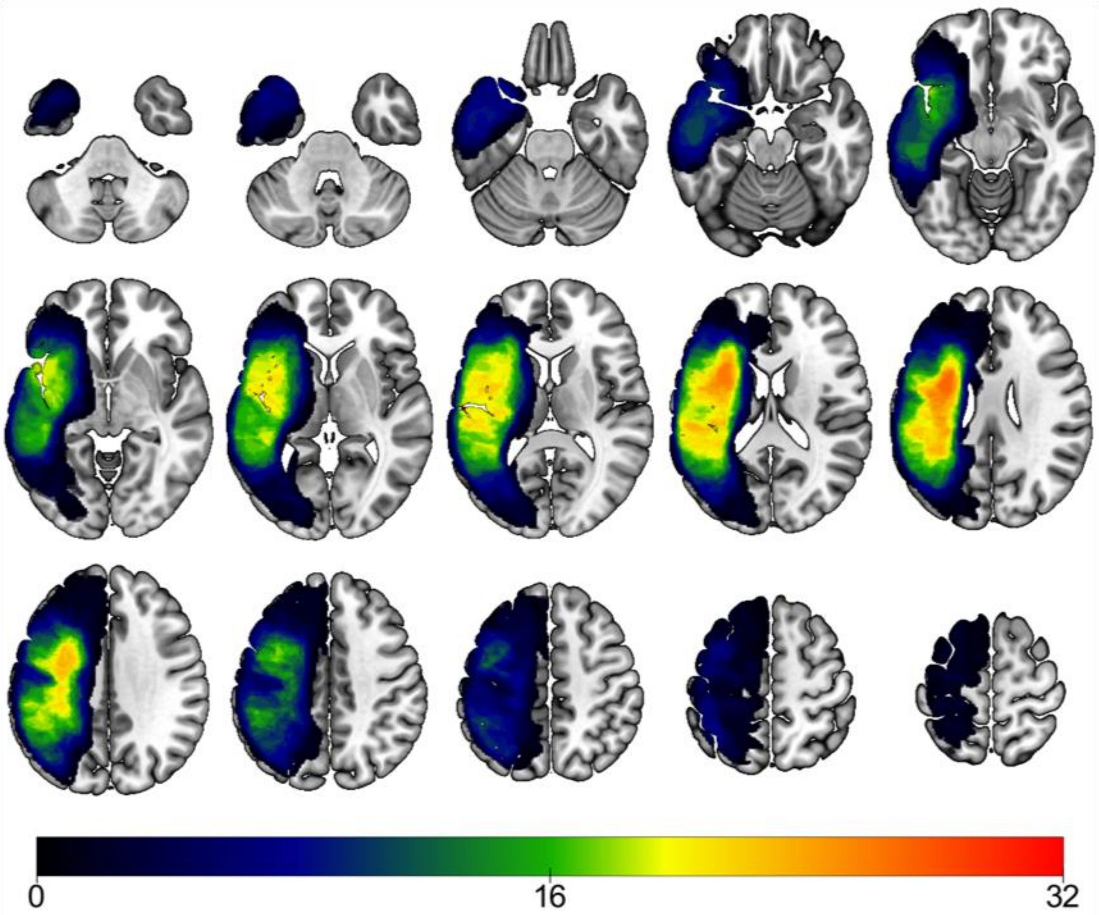

Supplementary Figure 1: Lesion Location: Lesion overlap of all participants (N = 32). The different colors represent a different amount of patients with lesions in that area.
